# Revolutionizing Restorative Dentistry: The Role of Polyethylene Fiber in Biomimetic Dentin Reinforcement—Insights from In Vitro Research

**DOI:** 10.3390/jfb16020038

**Published:** 2025-01-22

**Authors:** Álvaro Ferrando Cascales, Andrea Andreu Murillo, Raúl Ferrando Cascales, Rubén Agustín-Panadero, Salvatore Sauro, Carmen Martín Carreras-Presas, Ronaldo Hirata, Artiom Lijnev

**Affiliations:** 1 Department of Biomaterials Engineering, Faculty of Medicine, Universidad Católica de Murcia, UCAM, Campus de los Jerónimos, 135 Guadalupe, 30107 Murcia, Spain; rferrando@ucam.edu (R.F.C.); alijnev@ucam.edu (A.L.); 2 Faculty of Medicine, Universidad Católica de Murcia, UCAM, Campus Los Jerónimos, 135 Guadalupe, 30107 Murcia, Spain; aandreu87@alu.ucam.edu; 3 Prosthodontic and Occlusion Unit, Department of Stomatology, Faculty of Medicine and Dentistry, Universitat de València, 46010 Valencia, Spain; ruben.agustin@uv.es; 4 Dental Biomaterials and Minimally Invasive Dentistry, Department of Dentistry, University CEU Cardenal Herrera, C/Santiago Ramón y Cajal, s/n, Alfara del Patriarca, 46115 Valencia, Spain; 5 Department of Therapeutic Dentistry, I. M. Sechenov First Moscow State Medical University, 119146 Moscow, Russia; 6 Head of Esthetic Dentistry Program, School of Denstistry, Faculty of Biomedical Sciencies, Universidad Europea de Madrid, Tajo Street w/n, Villaviciosa de Odón, 28670 Madrid, Spain; carmen.martin2@universidadeuropea.es; 7 Department of Biomaterials and Biomimetics, New York University College of Dentistry, New York, NY 10010, USA; hirata@nyu.edu

**Keywords:** composite resin, biomimetics, fiber-reinforced, Ribbond, polyethylene fiber ribbond, long fibers

## Abstract

Recent advancements in biomimetic dentistry have introduced innovative materials designed to better simulate natural dentin. One such material is Ribbond^®^, long polyethylene fiber. It is particularly effective in absorbing and redistributing masticatory forces in teeth with substantial dentin loss. This review aims to analyze the literature on the biomimetic restorative technique using long polyethylene fiber and assess its benefits and indications relative to traditional cusp coverage restorations. Methods: A search was conducted in the PubMed database until March 2024. The authors selected in vitro studies that studied long polyethylene fiber as a dentin reinforcement. Results: From 247 potentially relevant articles, eighteen studies were included in the review. A detailed analysis of the reviewed literature was summarized into three principal sections involving the use of long polyethylene fiber in restorative dentistry. Conclusions: Long polyethylene fibers improve fracture resistance and promote favorable fracture modes, helping to mitigate the shrinkage forces in composite restorations. However, their clinical significance over traditional cusp coverage is unclear.

## 1. Introduction

Restorative dentistry has advanced significantly from basic mechanical retention to sophisticated adhesive techniques, driven by innovations in materials and adhesives [1,2]. Biomimetic dentistry represents a notable advancement, focusing on restoring a tooth’s original biomechanical integrity through materials that closely replicate the physical and mechanical properties of natural tissues. This approach ensures that restorative materials mimic the behavior of natural teeth under various conditions, such as thermal expansion and tensile strength, thereby preserving the integrity of dental tissues [3]. Key factors in restoring structurally compromised teeth include matching the elastic modulus and surface hardness of natural tissues to ensure even stress distribution and reduce fracture risk [3,4]. In that context, mimicking the amelodentinal junction is essential for harmonious load transfer and preventing the propagation of enamel cracks into the dentin [4].

The prognosis of restorations is significantly affected by the amount of remaining dental tissue, with structural factors, such as interaxial dentin, pulp chamber roof, marginal ridges, non-undermined cusps, and pericervical dentin thickness, serving as predictors of restoration success [5,6,7]. Greater structural compromise correlates with a higher risk of future catastrophic fractures due to reduced residual enamel and dentin, underscoring the importance of replicating natural interfaces to enhance the durability and effectiveness of dental restorations [8,9].

While fiberglass posts have traditionally been used to reinforce root canal-treated teeth, their effectiveness remains debated, and evidence is mixed [1,10]. Recent advancements in materials and adhesive systems aim to enhance the bonding strength of composite resins to dentin, improving adhesion to compromised dental structures. To address or redistribute tensile loads on composite restorations in teeth with substantial hard tissue loss, the incorporation of reinforcing fibers, especially glass and polyethylene fibers, has been proposed as a promising approach [11,12,13].

A leno-woven ultra-high molecular weight polyethylene (LWUHMW) fiber has been utilized to strengthen structurally compromised teeth. One commercially available example is the non-impregnated polyethylene fiber known as Ribbond. (RB), which undergoes cold gas plasma treatment to improve its chemical bonding with restorative materials. The classical use of these fibers includes periodontal splinting, Maryland direct bridges, endodontic posts and cores, stabilization of avulsed teeth, orthodontic retainers, and maintaining diastema closures [12]. Long polyethylene fibers (RB) are not new to the market; their increased use has been primarily driven by biomimetic restorative dentists.

In vitro studies have demonstrated that incorporating long polyethylene fibers can significantly improve the fracture resistance of heavily restored teeth and endodontically treated by acting as stress-absorbing layers that internally splint the tooth and reinforce the composite in multiple directions, effectively redistributing occlusal forces and preventing crack propagation [14,15]. Some authors denote the effectiveness in reducing adhesive stresses during polymerization and minimizing marginal microleakage, all without compromising the aesthetic results. Additionally, long polyethylene fibers possess a fail-safe mechanism that prevents catastrophic failure, allowing for a repairable tooth structure. However, several potential drawbacks have also been identified in studies, including the presence of porosities, adhesive failures, longer clinical application times, and handling difficulties [16,17,18,19]. Furthermore, there remains a lack of studies evaluating the effectiveness of polyethylene fibers in sclerotic or carious dentin, which, from a clinical standpoint, represents one of the most reasonable indications for their use, given that reinforcement is typically recommended for highly compromised teeth.

Overall, each material should be viewed as an additional tool in the clinician’s arsenal rather than a necessity. Clinicians should prioritize adhesion and strive to simplify procedures without compromising the quality or longevity of treatments.

The purpose of our narrative review is to provide clinicians with a comprehensive overview of the advantages offered by long polyethylene fiber reinforcement, contrasting these benefits with those of conventional protocols and trying to answer the arising question: does the integration of long polyethylene fibers into adhesive protocols used as a dentin reinforcement provide sufficient advantages to warrant its consideration as a new clinical approach?

## 2. Materials and Methods

The study protocol was registered on 1 October 2024 on the Open Science Framework (OSF) database (https://doi.org/10.17605/OSF.IO/QJ74A) under number CRD42021295212. The guidelines of the PRISMA-ScR 2018 statement were used to guide the reporting of this narrative review. Only in vitro studies were considered for this review.

### 2.1. Search Strategy

A thorough electronic literature search was performed on PubMed up to March 2024. Additionally, during the review process, the relevant literature was further gathered from the reference sections of the retrieved articles to supplement the information. The search utilized the following MeSH terms as keywords: “composite resin”, “biomimetics”, “fiber-reinforced” and “ribbond”. The final search was conducted using the following combination of keywords: “Composite resin AND Biomimetics AND fiber-reinforced OR ribbond”.

### 2.2. Eligibility Criteria

Scientific papers eligible for inclusion in the present review were published in English, peer-reviewed, and dated from 2019 to 2024. To present the most up-to-date and comprehensive evidence, this study incorporates the latest findings and recent advancements in the field. The selected articles were required to include the search terms in either the title or abstract. Preference was given to full-text published articles. Systematic reviews, meta-analyses, unpublished works, personal communications, background information, and advertisements were excluded from consideration.

### 2.3. Selection Process

Two investigators independently searched and screened the results using the agreed inclusion criteria: (a) in vitro laboratory studies; (b) related to long polyethylene fiber material; (c) comparison between properties of long polyethylene fiber and conventional particulate filler composite (PFC) resins; (d) published in a peer-reviewed journal; (e) reported manufacturer information; and (f) published in English.

Whenever it was not possible to make this determination, the full-text article was examined. Subsequently, all relevant articles were obtained, and those not meeting the inclusion criteria were excluded from the review.The selection and screening process was made using Microsoft Excel (16.93 version).

### 2.4. Data Synthesis

After omitting the duplicates/repetitive articles, a total of 18 full-text articles were studied and concluded for review, for that purpose EndNote 20.4 was used. For each article, the authors’ names, study setup, main results, and conclusion were reported (Table 1). Additionally, main findings, limitations, and controversies were summarized (Table 2).

## 3. Results

**Figure 1:**
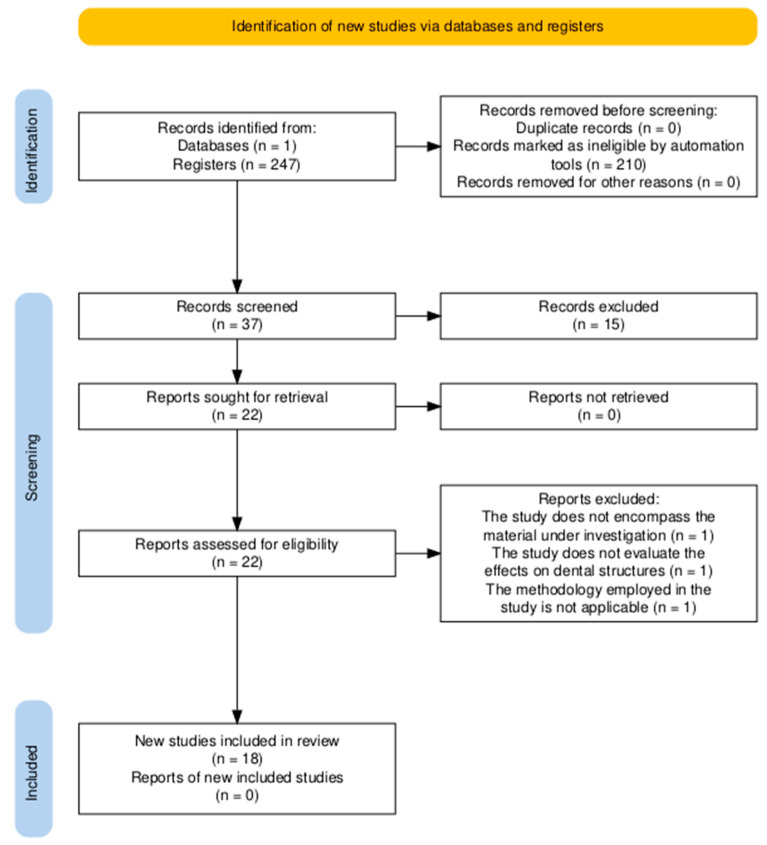
PRISMA flow diagram of the screening and selection process.

A total of 247 relevant articles were identified and screened by evaluating their titles and abstracts. Following a thorough assessment, 225 articles were excluded due to not meeting the inclusion criteria or being duplicates. Consequently, 22 articles were selected for their relevance to this review. After a full-text screening of text articles, three further studies were excluded due to methodological issues. Finally, 18 articles were included in our review for full-text analysis. Figure 1 shows the screening and selection process in a PRISMA flow diagram. 

**Figure 2:**
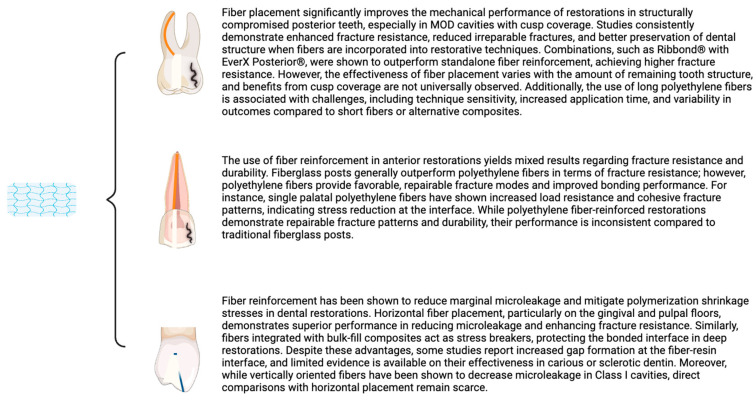
Summary of key points from each section analyzed in the results.

Nine studies evaluated the influence of fiber and/or different restorative modalities of structurally compromised posterior teeth; four articles analyzed the effect of fiber and gap formation and stress distribution; and five studies aborded restoration of anterior teeth with fibers (Figure 2). Seventeen included publications investigated the effect of fiber on extracted teeth, twelve on posterior premolars and molar teeth, five on anterior, and one study analyzed the effect of fiber reinforcement on resin composite molds.

## 4. Discussion

This comprehensive review aims to equip clinicians with an in-depth understanding of biomimetic restoration techniques utilizing long polyethylene fibers and to offer a clinical rationale for their application. A detailed analysis of the reviewed literature categorizes the examined characteristics into three principal sections. Each section provides a summary of current evidence on the topic, addresses existing limitations, and outlines potential directions for future research.

### 4.1. Influence of Fiber Placement on Mechanical Performance in Structurally Compromised Posterior Teeth

The loss of dental structure and the compromise of the remaining structure directly affect tooth strength [35]. Research shows that the involvement of marginal ridges is associated with structural compromise and weakening of the tooth [36]. In this context, most studies assess the design of MOD [5,14,22,26,28,30,32,33] and Class II [21,31] cavities to replicate compromised situations, frequently in combination with endodontic treatment [14,22,28,32]. However, the study by Sfeikos, Dionysopoulos, Kouros, Naka, and Tolidis [2] analyzed the situation in Class I cavities.

Most authors agree that fiber incorporation improves fracture resistance in restorations [5,21,22,26,28,32], cuspid deflection [30], and fatigue resistance [14]. However, some authors argue that its effectiveness may depend on the amount of remaining tooth structure [5].

Some researchers had explored the effectiveness of fibers when cusp coverage is incorporated [14,23], denoting that fiber incorporation and overall resistance benefits from cusp coverage [7], while Fildisi and Eliguzeloglu Dalkilic [23] found no significant changes when fibers were added to overlay or endocrown restorations. Moreover, polyethylene fibers were found to outperform glass fiber posts in terms of enhancing restoration strength and longevity [7].

Furthermore, authors such as Agrawal, Shah, and Kapoor [26] and Fildisi and Eliguzeloglu Dalkilic [23] observed that the incorporation of fibers reduces the frequency of irreparable fractures and favors the preservation of the dental structure. This implies that RB can play a pivotal role in maintaining the structural integrity of restorations.

Some authors also noted that the incorporation of short fibers improves mechanical properties, outperforming long fibers [14,22,33]. Soto-Cadena, Zavala-Alonso, Cerda-Cristerna, and Ortiz-Magdaleno [22] found that combining RB and EverX Posterior significantly enhances fracture resistance compared to using RB alone. This suggests that while RB can contribute to reinforcement, it may not be sufficient as a standalone material, emphasizing the importance of combining materials to achieve optimal results.

Recent studies evaluating the fracture resistance and failure modes of polyethylene fiber-reinforced restorations have not demonstrated significant improvements in the fracture resistance of teeth restored with these fibers. Surprisingly, a notable finding was the increased occurrence of adhesive failures at the tooth–restoration interface. Furthermore, the studies reported a concerning presence of porosities and defects at the fiber–resin interface, which could serve as potential sites for water accumulation and material degradation. These observations highlight critical challenges in the performance and resistance of teeth [17].

### 4.2. Impact of Fiber Reinforcement on Microleakage and Gap Formation in Dental Restorations

In studies, the predominant fiber placement orientation was horizontal. However, several authors specifically studied fiber orientation and found a correlation between fiber positioning relative to the cavity and the outcomes achieved. Albar and Khayat [33] observed that fibers oriented along the axial and gingival walls exhibited better fracture resistance results. Similarly, Agrawal, Shah, and Kapoor [26] noted that fibers placed horizontally on the gingival and pulpal floors showed improved fracture resistance, outperforming restorations without fibers. This suggests that horizontal fiber orientation is beneficial due to reinforcement when fibers are perpendicular to applied forces.

Conversely, Sfeikos, Dionysopoulos, Kouros, Naka, and Tolidis [2] observed that vertically placed fibers reduced microleakage, although no comparison was made with horizontal placement, and the cavity design was Class I. In contrast, Hasija, Meena, Wadhwa, and Aggarwal [31] found that fiber incorporation did not improve adaptation in compromised caries-affected dentin.

One of the challenges in restorative dentistry is minimizing the stress caused by polymerization shrinkage. Recently, bulk-fill composites, designed to be placed in a single increment of 4 mm thickness, have been introduced as a potential solution to streamline the restorative process and reduce polymerization shrinkage stress.

Several authors have studied the relationship between fiber placement and bulk-fill restorative materials. Sfeikos, Dionysopoulos, Kouros, Naka, and Tolidis [2] found that the addition of fibers reduces marginal microleakage regardless of the bulk-fill material or the application protocol. Similarly, Sadr, Bakhtiari, Hayashi, Luong, Chen, Chyz, Chan, and Tagami [27] demonstrated that the incorporation of fibers significantly reduces gap formation in the deepest areas of the cavity, also acting as a shrinkage stress breaker and protecting the bonded interface in deep dentin. Conversely, Deger, Ozduman, Oglakci, and Eliguzeloglu Dalkilic [30] found that fiber incorporation exhibited greater gap formation. Recently, another interesting study concluded that the placement of fibers on the pulpal floor or axial wall to minimize stress does not offer benefits over classical alternatives with conventional composite resin [33].

### 4.3. Analysis of Fiber Reinforcement and Fracture Resistance in Anterior Dental Restoration

Studies on fiber reinforcement in anterior dental restorations reveal varying effects on mechanical performance and fracture resistance. Some authors compared fiber reinforcement with fiberglass posts in anterior restorations [25,29]. Sreen, Abraham, Gupta, Singh, Aggarwal, Chauhan, Jala, and Mehta [29] observed that restorations with fiberglass posts required more force to fracture compared to those with RB, which demonstrated less resistance. This is consistent with findings that restorations with fiberglass posts exhibited greater fracture resistance than those with RB, regardless of fracture type [25]. Both studies suggest that traditional fiberglass posts may provide superior mechanical support compared to long polyethylene fibers in enhancing the strength of anterior restorations.

In contrast, other authors found positive results for polyethylene fibers in anterior restorations [20,34]. Jadhav, Mittal, Shinde, Al-Qarni, Al-Obaid, Abullais, Cicciu, and Minervini [34] demonstrated that composites with a single palatal polyethylene fiber achieved higher load resistance and primarily fractured in the remaining tooth structure, indicating reduced stress at the interface and improved bonding. Similar research observed that composites reinforced with polyethylene fibers mostly exhibited cohesive fractures in incisal and mesioincisal restorations, suggesting enhanced fracture resistance [20]. Both studies support the efficacy of polyethylene fibers in improving the mechanical performance and durability of anterior restorations compared to traditional reinforcement methods.

### 4.4. Potential Barriers to Clinical Adoption of Long Polyethylene Fibers

Another important aspect to consider is the potential barriers to the clinical adoption of polyethylene fibers. Some studies have reported that the complex application techniques and restoration designs involving RB fiber did not result in significant improvements in clinical outcomes but instead introduced notable handling difficulties [16,18]. The most recent randomized clinical trials addressing this issue have concluded that the application of RB-reinforced resin composite restorations can be both expensive and time-consuming, particularly in cases of moderate tooth loss [19].

## 5. Limitations and Recommendations for Future Research

The main limitation of this review is that it is based solely on in vitro studies and mainly on mechanical performance. In our opinion, these in vitro studies are necessary to provide information to clinicians and industries about the expected clinical behavior of a material, and it is the previous necessary step for a clinical trial of a material. However, we acknowledge the importance and necessity of clinical trials with long-term follow-up to evaluate the potential benefits of polyethylene fiber-reinforced restorations over traditional cusp-coverage restorations. These trials are essential to assess the challenges associated with handling procedures, time management, and the cost-benefit of incorporating polyethylene fibers into routine clinical practice.

## 6. Conclusions

Within the limitations of this review, long polyethylene fibers appear to enhance overall fracture resistance and promote favorable fracture modes. However, their clinical significance compared to traditional cusp coverage methods remains uncertain. Long polyethylene fibers seem to offer protection to the hybrid layer by mitigating polymerization shrinkage, especially from low-filled resin composites; however, they can incorporate porosities and defects at the fiber–resin interface. Conversely, short fiber-reinforced composites have shown promising results when compared with long polyethylene fibers. The learning curve and reproducibility remain potential drawbacks for clinicians to incorporate long polyethylene fibers, The above findings do not support the systematic integration of long polyethylene fibers into adhesive protocols, highlighting the need for further studies to fully elucidate the potential advantages of polyethylene fiber-reinforced restorations over conventional approaches in direct restorative dentistry and to comprehensively evaluate the cost-benefit of incorporating fibers into clinical practice.

## Figures and Tables

**Table 1:** Details of the included publications.

Author	Study Type	Study Setup	Main Results	Conclusion
Patnana, et al. [20]	In vitro	42 permanent incisors Group I: restoration in incisal fracture. Group II: restoration in mesioincisal fracture. Each of these two groups is later subdivided into three subgroups based on the restorative material used: A (particle composite restorations), B (fiber-reinforced composite), and C (polyethylene fiber-reinforced composite restorations). Three fracture patterns were recorded: cohesive, adhesive, or mixed.	A significant association was found between glass fiber-reinforced restorations and a fracture pattern in both incisal and mesioincisal areas.	Glass fiber-reinforced and polyethylene fiber-reinforced restorations exhibited better fracture resistance properties than traditional particle-filled composites in incisal and mesioincisal restorations.
Albar and Khayat [21]	In vitro	60 molars/premolars prepared with class II cavity. Group I: direct composite restoration. Group II: composite restoration reinforced with fibers placed in the axial wall of the proximal cavity. Group III: composite resin restoration reinforced with polyethylene fibers placed in the gingival floor of the proximal cavity. Group IV: composite with fibers placed in both the axial wall and the gingival floor of the proximal cavity.	Group IV had the highest average load resistance (148.74 MPa), followed by group II (140 MPa), group III (136.34 MPa), and group I (130.08 MPa).	Based on the results of the study, it was found that Class II cavities restored with composite reinforced with polyethylene fibers increase the fracture resistance compared to the conventional non-reinforced ones. The greatest difference was found when the fibers were settled in both the axial wall and the gingival floor, although this is not statistically significant.
Soto-Cadena, et al. [22]	In vitro	40 premolars with (mesio occlusal distal) MOD preparations and endodontic treatment. Group I (short fiber reinforced composite) SFRC (EverX Posterior GC, Tokyo, Japan).) followed by conventional composite resin. Group II with polyethylene-reinforced fibers (PRF) (Ribbond, Ribbond Inc., Seattle, WA, USA) followed by conventional composite resin. Group III restored with the PRF + SFRC combination followed by conventional composite resin. Control group (intact teeth).	The restoration with PRF + SFRC provided the highest fracture resistance (288.2 ± 73.5 N). The restoration with RB alone obtained the lowest values (192.4 ± 25.4 N), which showed a statistically significant difference with those of SFRC and PRF + SFRC. All fracture patterns were favorable.	Reinforcing endodontically treated premolars with MOD cavities with fibers from RB followed by a glass fiber reinforced resin layer and a conventional composite resin restoration improves fracture resistance and may be suitable for direct coronal restoration of large posterior cavities in high stress areas.
Volom, Vincze-Bandi, Sary, Alleman, Forster, Jakab, Braunitzer, Garoushi and Frater [7]	In vitro	120 third molars with MOD preparations and endodontic treatment SFC group+ CC: discontinuous short fiber reinforced composite discontinuous with cuspid coverage (CC). PFRC group: SFC without cuspid coverage. PFRC + CC group: transcoronal fixation with continuous polyethylene fibers with CC. GFRC group: continuous glass FRC post without CC. Group GFRC+CC: continuous glass FRC post with CC. Control SFC group (control).	The PFRC + CC group was characterized by significantly longer survival compared to all groups in the control group. In contrast, the GFRC group showed significantly lower survival compared to all groups except the SFC + CC group. The control group (SFC) showed statistically higher survival than the SFC+CC group and the GFRC group but did not differ significantly from the rest of the groups in terms of survival.	Direct restorations using continuous FRC systems (in the form of polyethylene fibers or FRC post) to restore RCT (endodontically treated) molar cavities with MOD cavities performed better in terms of fatigue resistance when cuspid coverage (CC) was performed, compared to the same FRC restorations without CC. Teeth restored with SFC performed better without CC compared to those groups with coverage.
Fildisi and Eliguzeloglu Dalkilic [23]	In vitro	65 molars. Group IN (intact teeth). Group E (endocrown). Group ER (endocrown + fibers). Group O (overlay). Group OR (overlay + fibers). RB was inserted at the base of the pulp chamber in the ER and OR groups.	Group E showed significantly lower fracture toughness values than other groups. No statistically significant differences were found between the other groups. Most of the unfavorable fractures were found in groups E and O. The overlay restorations showed higher fracture toughness values than the endo crown restorations.	Although fiber insertion did not improve the fracture strength of indirect restorations, it did reduce the frequency of the irreparable fracture mode. Overlay restorations and fiber application are more advantageous in preserving the durability of endodontically treated teeth.
Sfeikos, Dionysopoulos, Kouros, Naka and Tolidis [2]	In vitro	120 molars. 12 groups (n = 10) according to type of restorative material (Filtek Z550 (3M), Beautifil II LS (SHOFU INC., Kyoto, Japan) or Beautifil Bulk Restorative (SHOFU). The use or absence of reinforcement fibers (RB). The restorative technique applied (incremental or bulk fill).	The type of material, the use of RB fibers, and the type of restoration technique significantly affected marginal microleakage. The placement of RB fibers decreased marginal microleakage of all restorative materials, irrespective of restorative technique, in a range of 31.2–81.4%. In the non-RB groups, between materials, there was no significant difference in microleakage when teeth were restored with the incremental technique.	The use of polyethylene fibers in the wallpapering technique in Class I composite restorations can be an effective method to reduce marginal microleakage, regardless of the technique or restorative material selected.
Kinikoglu, et al. [24]	In vitro	120 maxillary permanent central incisors. Group 1: positive control group. Group 2: negative control group. Group 3: bulk fill group. Group 4: SFRC group. Group 5: composite resin group. Group 6: polyethylene fiber group.	A significant difference was detected between the groups, with the positive control group. Positive control group showed the highest mean fracture strength. The SFRC group had significantly higher values than the bulk filler, polyethylene fiber, fluid composite resin, and negative control groups.	SFRC has a relatively high fracture toughness compared to other materials used in restorative endodontic procedures. The use of SFRC improved the fracture resistance of immature permanent teeth.
Khurana, et al. [25]	In vitro	60 maxillary incisors. Group A: labiopalatal fractures. Group B: palatolabial fractures. They were divided into subgroups: Subgroup I: fiber from Ribbond was used. Subgroup II: EverStick (GC, Tokyo, Japan). post was used. The fractured fragments were repositioned with resin.	The labiopalatal fracture pattern of group A showed significantly greater resistance to fracture than the palatolabial fracture pattern of group B in both subgroups. Similarly, subgroup II with the Everstick post exhibited significantly greater resistance to fracture than subgroup I with RB in groups A and B.	The remaining tooth structure should be preserved, as it offers significant resistance to fracture. The EverStick post showed significantly high fracture resistance compared to the other FRC post (RB) and may be a promising alternative to conventional core post systems and other FRC post systems.
Agrawal, et al. [26]	In vitro	120 maxillary premolars. Group I: posterior G-aenial (GC, Tokyo, Japan). Group II: posterior G-aenial (GC) + horizontal placement of RB on the pulp/gingival floor. Group III: posterior G-aenial (GC) + horizontal placement of RB only on the pulp floor. Group IV: posterior G-aenial (GC) + vertical placement of RB on the gingival/pulpal floor. Group V: posterior G-aenial (GC) + particles of RB. Group VI: EverX posterior (GC, Tokyo, Japan).	The placement of the fibers significantly increased the fracture resistance. The highest fracture resistance was presented by group II, followed by group III, group IV, group V, and group VI. Group I (without fiber) showed the lowest fracture resistance. Repairable fractures occurred most frequently in group II, followed by group VI, and group I had the lowest frequency.	The horizontal orientation of the polyethylene fibers in both locations (pulp and gingival floor) of MOD cavities provides the greatest resistance to fracture in maxillary premolars and repairable fractures.
Sadr, et al. [27]	In vitro	Composite resin molds (3 mm wide and 4 mm deep). (1) SureFil (Dentsply, York, PA, USA) SDR (Dentsply) bulk-placed fluid (BLK). (2) SDR (Dentsply) placed in two unequal layers (INC). (3) SDR (Dentsply) placed after an increment of SDR (Dentsply) placed next to wetted polyethylene fibers (RBUltra) in the cavity floor (FRC). Control group, SDR (Dentsply) without bonding agent.	The sizes of the breccia were different among the groups. The largest were observed on the floor of the cavity of the control group, followed by BLK and INC. In FRC, no gaps were observed in the cavity floor, but some were observed between the layers.	The formation of voids in the cavity floor was significantly reduced by the placement of a layer of a fiber-reinforced layer at the base of the deep preparations. This reinforcement acts as a shrinkage stress breaker and protects the interface.
Verma, et al. [28]	In vitro	80 mandibular premolars. Group 1: intact teeth (control). Group 2: MOD (unfilled). Group 3: MOD restored with composite resin. Group 4: MOD restored with Cention N,(Ivoclar Vivadent, Schaan, Liechtenstein). Group 5: 10 mm fiber post with composite resin. Group 6: 5 mm fiber post with composite resin. Group 7: RB on occlusal surface and composite resin, Group 8: horizontal fiber post with composite resin.	The highest fracture resistance was presented by the control group (Group 1) followed by group 7, group 8, group 5, group 6, group 3, group 4, and finally, the lowest resistance was presented by group 2.	It was observed that the best fracture resistance was of intact teeth, followed by RB on the occlusal surface after endodontic treatment, and the lowest fracture resistance was found in unfilled MOD. The RB is good for occlusal protection of the tooth compared to other materials.
Sreen, et al. [29]	In vitro	48 maxillary central incisors. Group 1: control group. Group 2: repositioning of the fragment followed by placement of two external vertical grooves on the labial surface and restoration with polyethylene fibers and hybrid composite. Group 3: repositioning of the fragment followed by two external vertical grooves filled with fiber posts and composite. Group 4: repositioning of the fragment followed by circumferential chamfer at the fracture line and restoration with composite.	The highest values of the force required to fracture were observed in the fiber post group and the lowest in the RB group. The fiber post group had results with statistically significant differences compared to the RB and chamfer preparation groups.	The force required to fracture the fiber post group was closest to that of the intact teeth, followed by the chamfer and RB groups, respectively.
Deger, et al. [30]	In vitro	32 maxillary premolars prepared with Class II MOD. Group I: Filtek Z250 (3M, St Paul, MN, USA)) group (control)/microhybrid composite site. Group II: EST group/low viscosity bulk fill + microhybrid composite. Group III: NOV/low viscosity nanofiber reinforced low viscosity composite + microhybrid composite. Group IV: RB/polyethylene fiber + microhybrid composite.	No significant differences in volumetric cuspid deflection and fracture toughness were observed between any of the groups tested. The RB group showed significantly higher space deformation values than all other groups tested. There were no significant differences in gap formation for other groups tested.	Different interlayer materials do not affect cuspid deflection and fracture toughness, while the use of polyethylene fiber exhibits significantly higher gap formation compared to other groups tested under Class II composite restorations.
Hasija, et al. [31]	In vitro	Premolars and molars. Group I: A2 microhybrid universal composite resin with a 1-bottle adhesive system. Group II: RBfibers added to the same composite used in group I.	The results showed that there was a definite gap along the interface between the caries-affected dentin and the composite material in both groups. The largest gap was found in group II compared to group I.	Within the limitations of the study, the addition of polyethylene fiber in the composite material does not improve marginal adaptation, particularly in the affected dentin at the gingival margin.
Balkaya, et al. [32]	In vitro	120 mandibular premolars. G1: negative control (intact). G2: positive control (unrestored). G3: Filtek Z550(3M). G4: Filtek bulk fill restorative (FBR) (3M). G5: SDR (Dentsply) + Filtek Z550 (3M). G6: EverX Posterior+ Filtek Z550 (3M). G7: RB + FBR. G8: RB + SDR (Dentsply) + Filtek Z550 (3M).	The negative control group showed the highest mean fracture toughness compared to the rest of the groups, and the positive control group was the lowest. The RB + FBR group and the RB + SDR (Dentsply) + Filtek Z550 (3M) group showed a higher mean fracture toughness than the rest of the study groups but without much significant difference between these two groups. The most predominant fracture pattern was restorable but with no significant difference between groups.	The type of coronal restorative material could affect the fracture resistance of teeth with moderate hard tissue loss undergoing simulated regenerative endodontic treatment. The use of RB, in combination with composite resin, could be recommended as a reinforcement material in immature teeth undergoing regenerative endodontic treatment.
Albar and Khayat [33]	In vitro	72 posterior teeth. Group 1: conventionally restored with a nanohybrid composite resin (control group). Group 2: ACTIVA BioACTIVE-Restorative (Watertown, MA, USA) and Liner as a dentin substitute and covered with a nanohybrid composite. Group 3: composite resin EverX Posterior covered with nanohybrid composite. Group 4: RB placed on both axial walls and cavity floor, covered by a nanohybrid composite. Group 5: RB placed on both axial walls and on the floor of the cavity covered by ACTIVA Bio ACTIVE-Restorative and Liner as a dentin substitute and nanohybrid composite. Group 6: RB placed on both axial walls and cavity floor, covered with EverX Posterior composite resin and nanohybrid composite.	The highest maximum load was exhibited by group 3 with the EverX Posterior composite resin, followed by group 4, group 6, group 1, group 2, and group 5. A statistically significant difference was shown between the groups.	Within the limitations of the study, it can be concluded that higher peak load strength (statistically significant) can be achieved by reinforcing MOD nanohybrid composite resin restorations with EverX Posterior.
Jadhav, et al. [34]	In vitro	90 maxillary incisors. Group I: composite resin. Group II: composite resin and single fiber palatally placed. Group III: composite resin and two fibers palatally placed.	The data showed that group II presented the highest load values, followed by group I and group III.	The addition of FRC to the incisal composite build-up increased the strength of the restoration. The one reinforced with a single fiber showed higher load-capacity compared to two fibers or none.
Zotti, Hu, Zangani, Albanese and Paganelli [5]	In vitro	20 upper and lower molars. (1) Cavity MOD of 5 mm depth with residual interaxial dentin without RB. (2) Cavity MOD of 5 mm depth with residual interaxial dentin restored with RB fibers. (3) Cavity MOD of 5 mm depth without restored residual interaxial dentin without RB. (4) Cavity MOD of 5 mm depth with no residual interaxial dentin restored with RB.	There was a statistically significant difference between groups I and II in the loading force required to fracture. Contrary, there was no statistically significant difference between groups III and IV. Groups I and II had the lowest number of non-restorable fractures.	The application of fiber in MOD cavities appears to be more effective in terms of strengthening where the cavities have interaxial dental tissue.

**Table 2:** Summary of main findings, limitations, and controversies.

	Main Findings	Limitations	Controversies
Influence of fiber placement on mechanical performance in structurally compromised posterior teeth	Fiber placement enhances fracture resistance, particularly in MOD cavities, when combined with cusp coverage ([7,26,33]).	Effectiveness varies depending on remaining tooth structure, and cusp coverage benefits are inconsistent ([5,23]).	Some studies favor polyethylene fibers for resistance, while others report short fibers or alternative composites are equally effective ([14,22,33]).
	RB combined with materials like EverX Posterior showed the highest reinforcement ([22,26]).	In vitro studies dominate, limiting applicability to real-world clinical scenarios.	Debate over cost-effectiveness and clinical significance compared to traditional alternatives ([16,18]).
	Fiber-reinforced composites reduced irreparable fractures and preserved more dental structures ([23,26]).	Long application times and technique sensitivity limit their reproducibility ([16,23]).	No consensus on whether fibers significantly outperform other conventional reinforcement materials ([18,33]).
Impact of fiber reinforcement on microleakage and gap formation in dental restorations	Horizontal fiber placement (gingival/pulpal floors) reduces microleakage and enhances fracture resistance ([26,33]).	Contradictory results on gap formation: some studies show reduced gaps, while others report larger gaps at the fiber–resin interface ([30,31]).	Debate over the effectiveness of fiber reinforcement in bulk-fill composites versus conventional alternatives ([30,33]).
	Bulk-fill composites combined with fibers decreased polymerization shrinkage stress, protecting bonded interfaces ([27]).	Limited evidence for performance in clinically compromised dentin, such as carious or sclerotic dentin ([31]).	Studies lack a unified methodology for testing fiber placement, making comparisons challenging ([31,33]).
	Vertically oriented fibers reduced microleakage in Class I restorations ([2]).	No direct comparison of vertical versus horizontal placement in most studies ([2]).	Effectiveness in reducing shrinkage stresses remains inconsistent across fiber orientations ([2,33]).
Analysis of fiber reinforcement and fracture resistance in anterior dental restorations	Fiberglass posts outperformed polyethylene fibers in fracture resistance of anterior restorations ([25,29]).	Limited evidence supporting polyethylene fibers in anterior teeth restorations ([25]).	Fiberglass posts are preferred in some cases, but polyethylene fibers provide favorable fracture modes in others ([20,34]).
	Single palatal polyethylene fibers improved fracture resistance and reduced interface stress, showing cohesive fractures ([20,34]).	Lack of long-term clinical data to confirm durability of fiber-reinforced anterior restorations ([25]).	Mixed evidence on whether polyethylene fibers match the load resistance of fiberglass posts ([25,29]).
	RB-reinforced restorations showed repairable fracture patterns, preserving the remaining tooth structure ([20]).	In vitro studies dominate, limiting their application to clinical practice.	Contradictory results on whether polyethylene fibers enhance anterior restoration durability ([20,25,29]).

## Data Availability

No new data were created or analyzed in this study. Data sharing is not applicable to this article.
